# What failure in collective decision-making tells us about metacognition

**DOI:** 10.1098/rstb.2011.0420

**Published:** 2012-05-19

**Authors:** Bahador Bahrami, Karsten Olsen, Dan Bang, Andreas Roepstorff, Geraint Rees, Chris Frith

**Affiliations:** 1 UCL Institute of Cognitive Neuroscience, University College London, Alexandra House, London WC1N 3AR, UK; 2 Interacting Minds Project, Institute of Anthropology, Archaeology, Linguistics, Aarhus University, and Centre of Functionally Integrative Neuroscience, Aarhus University Hospital, 8000 Aarhus C, Denmark; 3 Department of Experimental Psychology, University of Oxford, Oxford OX1 3UD, UK; 4 Wellcome Trust Centre for Neuroimaging, Institute of Neurology, University College London, London WC1N 3BG, UK

**Keywords:** metacognition, collective decision-making, signal detection, cooperative behaviour, feedback, confidence

## Abstract

Condorcet (1785) proposed that a majority vote drawn from individual, independent and fallible (but not totally uninformed) opinions provides near-perfect accuracy if the number of voters is adequately large. Research in social psychology has since then repeatedly demonstrated that collectives can and do fail more often than expected by Condorcet. Since human collective decisions often follow from exchange of opinions, these failures provide an exquisite opportunity to understand human communication of *metacognitive confidence*. This question can be addressed by recasting collective decision-making as an information-integration problem similar to multisensory (cross-modal) perception. Previous research in systems neuroscience shows that one brain can integrate information from multiple senses nearly optimally. Inverting the question, we ask: under what conditions can two brains integrate information about one sensory modality optimally? We review recent work that has taken this approach and report discoveries about the quantitative limits of collective perceptual decision-making, and the role of the mode of communication and feedback in collective decision-making. We propose that shared metacognitive confidence conveys the strength of an individual's opinion and its reliability inseparably. We further suggest that a functional role of shared metacognition is to provide substitute signals in situations where outcome is necessary for learning but unavailable or impossible to establish.

## 1. Introduction

In *The extraordinary and popular delusions and madness of crowds*, Charles Mackay [1] chronicled a colourful and prolific history of humankind's collective follies.^1^ Mackay's decision to doubt and re-examine the popular belief that ‘two heads are better than one’ has since then guided numerous disciplines interested in human collective decision-making from political sciences to economics and social psychology. Mackay's negative revisionism was preceded by a wave of optimistic trust in mass decisions initiated by the Marquis de Condorcet [2], a mathematician and political philosopher of the French revolution. Condorcet's jury theorem elegantly proved that a simple ‘democratic’ majority vote drawn from the aggregated opinions of individual, independent and fallible (but not totally uninformed) lay-people provides near-perfect accuracy if the number of voters is adequately large [2].

At a local livestock fair in Plymouth, early in the twentieth century, Francis Galton [3] found strong empirical support for Condorcet's theoretical proposition. At a weight-judging contest, participants estimated the weight of a chosen live ox after it had been slaughtered and dressed. Participants entered the competition by privately writing their estimate on a ticket and submitting it to the fair organizers. The winner was the one who submitted the most-accurate estimate. After the competition, Galton collected the approximately 800 submitted tickets and demonstrated in a paper [3] that, indeed, the simple average of the estimates of the entire crowd was even more accurate than the winner. The most striking aspect of this finding was that the majority of participants had very little specialized knowledge of butchery; yet, their contribution to the average opinion outperformed the best expert opinion. Theoretically and empirically, masses ruled supreme. So, can we dismiss Mackay's [1] worries? Definitely not!

A large body of work in political sciences and social psychology has examined collective decision-making and, indeed, numerous examples of collective failure have been discovered [4]. Indeed, this research clearly shows that Condorcet's assumption about the independence of individual opinions—which was neatly satisfied in the weight-judging contest—is often not applicable to real-world situations of collective behaviour [5]. However, by carefully identifying the determinants of collective failure, we can use Mackay's [1] insight, that collective benefit is the exception rather than the rule, to better understand the nature of human social interaction. To rephrase, one could ask which features of interpersonal communication and/or interaction contribute to collective failures.

In this paper, we first review previous work that has addressed this question by recasting collective decision-making as an ‘information integration’ problem similar to multisensory (cross-modal) perception. In multisensory perception, the observer combines information from different sensory modalities (e.g. vision and touch) taking into account the reliability (or variance) of each modality such that the multisensory decision is more strongly influenced by the sensory modality with the higher reliability (i.e. lower variance) [6–8]. By analogy, collective decision-making also requires combining information but from different observers. We argue that establishing the reliability of this information constitutes an integral part of information integration at the collective level.

Using collective decision-making in the perceptual domain as a framework, we describe and compare two models of how we communicate and integrate our individual perceptions and their reliability [9,10]. Both models posit that observers convey the reliability of their individual perceptions by communicating their *confidence* in their perceptual decisions, i.e. their metacognitive awareness of their perceptual decisions. However, the models make very different assumptions about the exact content of the communicated confidence and the computational strategy by which observers combine them to arrive at a collective decision. We will review the predictions of each model and assess them in the light of the existing literature. We will also present new empirical data revealing further features of collective perceptual decision-making by confidence sharing. We will place the findings from collective perceptual decision-making in the wider context of group decision-making [4] and discuss a possible functional social role for metacognitive awareness. Finally, we will briefly compare and attempt to connect our current understanding of metacognition at the levels of brain mechanism, individual behaviour and social interaction.

## 2. Collective perceptual decision-making

**Figure 1.**
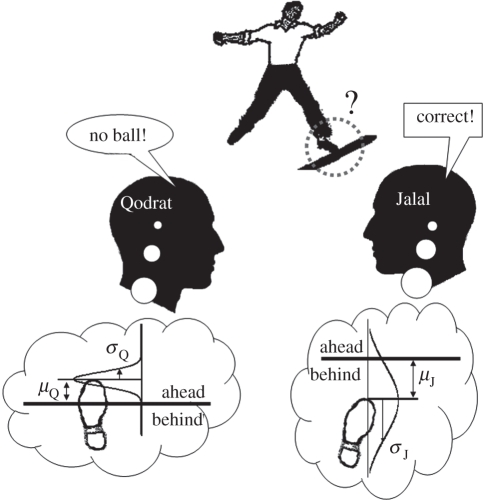
Two cricket umpires, Qodrat and Jalal, disagree about whether the bowler crossed the line. The low-quality image depicting the bowler was intentionally constructed to indicate the perceptual noise. Each umpire's individual decisions are based on his respective noisy perceptual representation, which we model as a Gaussian distribution. The figure is inspired by Ernst [11].

Qodrat and Jalal (figure 1) are two cricket umpires.^2^ The bowler (figure 1, top) has just made his run and bowled the ball; but the two umpires disagree about whether his foot crossed the line or not. Although Qodrat has announced a ‘no ball’, Jalal contends that there was no such error. Let us stop here and examine the situation. We can think of each umpire's visual perception of the events as represented in the brain by a normal distribution with a mean (*μ*_Q_ for Qodrat and *μ*_J_ for Jalal) and a standard deviation (*σ*_Q_ and *σ*_J_). This normal distribution could correspond to, for example, the firing pattern of neurons in each umpire's early visual cortex. The umpire's decision about whether the bowler's foot landed ahead of (e.g. Qodrat, *μ*_Q_ > 0) or behind (e.g. Jalal, *μ*_J_ < 0) the line is given by the signed mean of the distribution. The standard deviation of each distribution relates to the noisiness of the umpire's perception. As such, a reliable percept would be characterized by a large mean (e.g. Jalal) and a small standard deviation (e.g. Qodrat). But how do Qodrat and Jalal resolve their disagreement and come to a joint decision? The simple formulation of the situation given above is the basis of two recent models [9,10] of collective perceptual decision-making.

Sorkin *et al*. [10] proposed that, by communicating their confidence in their perceptual decision, the umpires are in fact communicating their, respective, *μ* and *σ* separately and distinctly to one another. As we will see further below, the distinctness of these two pieces of information is a critical feature of this model. To make an optimal collective decision (i.e. to minimize the chances of error given each umpires’ decision noise), the two umpires (i.e. the group) somehow evaluate the term 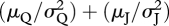 and take its sign as their joint decision. Defining perceptual sensitivity (*s*) as inversely proportional to standard deviation (such that *s* = *k*/*σ*; where *k* represents a constant term; see equation (3.3) below for the exact definition of slope, the group's sensitivity, *S*_group_, is then expected to be:2.1
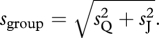


In a standard sensory signal-detection task performed by individuals and groups in separate experiments, Sorkin *et al*. [10] showed that groups achieved a robust collective benefit over and above the sensitivities of the constituent individuals as measured when these individuals performed the task in isolation. Their model was able to predict the collective benefits accrued by the groups. Interestingly, their model could readily be extended to groups larger than two people. However, as group size expanded, group performance did not improve as fast as predicted by the model, indicating that, perhaps, different group dynamics may be at work as group size increases.

Sorkin *et al*.'s [10] model is conceptually identical to the model used in multisensory perception research to describe how information from different sensory modalities, such as touch and vision, are combined within the brain of one observer [6,8,13]. That dyads performed as well as equation (2.1) would lead to the uncomfortable conclusion that communication between brains is as reliable and high fidelity as communication within the same brain. Moreover, this formulation implies that groups would *never* do worse than individuals. Recalling the case of Condorcet, Mackay and Galton, once again, groups seemed to be doing much better (theoretically and empirically) than common sense would suggest.

Nearly a decade later, Bahrami *et al*. [9] performed an experiment almost identical to that of Sorkin *et al*. [10], but they made a different assumption about the content of the information communicated between individuals. Noting that a reliable decision (figure 1) is one based on a large mean and a small standard deviation, they suggested that Qodrat's communicated confidence in his decision could be defined as the ratio *μ*_Q_/*σ*_Q_. The magnitude of this signed ratio would correlate with the probability that Qodrat has made the right decision. The collective decision could then simply be defined as the sign of the sum of shared confidences (*μ*_Q_/*σ*_Q_) + (*μ*_J_/*σ*_J_), giving the group sensitivity by2.2
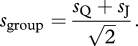


Bahrami *et al*. [9] dubbed this model the Weighted Confidence Sharing (WCS) model. Comparison with Sorkin *et al*.'s [10] model shows that when sensitivities are similar (i.e. *σ*_J_ = *σ*_Q_), the two decision boundaries 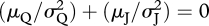 and 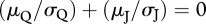 become identical and the outcome is equivalent to that seen in multisensory perception. When the individual sensitivities are different (say, *s*_Q_ > *s*_J_), however, the two models diverge in their predictions. To demonstrate this, if we rewrite equation (2.2) as2.3
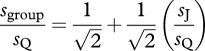


we can see that the expected collective benefit (*s*_group_/*s*_Q_; i.e. group sensitivity relative to its more sensitive member) is a linear function of the similarity between group members’ sensitivities (*s*_J_/*s*_Q_). This linear relationship means that if Jalal's sensitivity is no better than approximately 40% of Qodrat's (*s*_J_/*s*_Q_ < 2^1/2^−1 ≈ 0.4), then—in sharp contrast to Sorkin *et al*.'s [10] model—this model predicts that Qodrat and Jalal together should do worse than the more sensitive observer (Qodrat) alone (*s*_group_ < *s*_Q_).

**Figure 2.**
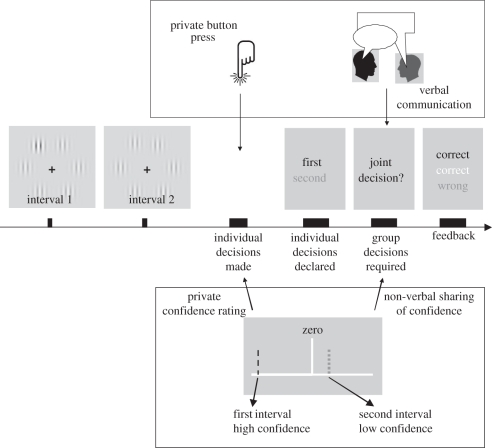
Stimuli, task and modes of communication. Each trial consisted of two observation intervals followed by private decisions by each participant. In the verbal communication mode (top box), participants indicated their individual decision by a button press. In the non-verbal mode (bottom box), participants reported the target interval by dragging a marker to the left (first) or right (second) of the centre and indicated their confidence by the distance of the line from the centre. Individual decisions were then announced, and in cases of disagreement, participants either talked to each other (top) or saw each others’ confidence rating (bottom) in order to reach a joint decision. Then one of the observers (indicated by the colour of the sentence ‘joint decision?’) announced the dyad decision. Grey, black and white shades correspond to blue, yellow and white colour codes that were used in the experiments to indicate the participant using the keyboard, the one using the mouse and the dyad, respectively.

Bahrami *et al*. [9] tested dyads in a simple perceptual decision-making task that involved visual contrast discrimination (figure 2). In every trial, individuals first made a private decision about a briefly viewed stimulus. If their private decisions disagreed, they were asked to negotiate a joint decision. When dyad members had similar sensitivities, dyad decisions were more accurate than those of the better individual. However, if one observer was much less sensitive than the other, the dyad failed to outperform the better member; cf. experiment 2 in the study of Bahrami *et al*. [9]. Importantly, group performance in these latter situations was markedly worse than expected from equation (2.1) but not statistically different from the predictions of equation (2.2). Mackay's intuition had once again proved useful: examination of collective failures suggested that perceptual decisions and their reliability are not spontaneously communicated separately but instead together in the form of a ratio. If what Qodrat communicates is the ratio (*μ*_Q_/*σ*_Q_), then Jalal (i.e. the recipient) will be unable to resolve (*μ*_Q_) and (*σ*_Q_) from one another. Consequently, the process of interpersonal communication involves information loss. But is there a way to avert or reduce this loss of information?

Previous research suggests that failures of communication are not solely owing to noise and random errors [14]. A number of systematic egocentric biases that impair communication have been identified on both sides of a verbal exchange. When communicating their internal intentions verbally, people often overestimate the clarity of their communicated message, as if their internal states were readily evident, indeed *transparent*, to their addressee [15]. Similarly, an egocentric bias afflicts the addressee: listeners often interpret the meaning of what they are told from their own (rather than the speaker's) perspective [16].

We hypothesized that if the collective failures observed by Bahrami *et al*. [9] were a consequence of egocentric biases that plague face-to-face verbal communication [14], then providing a non-verbal, scalar system for participants to share information may remove or reduce these egocentric biases and in turn improve collective decision-making; especially when observers have vastly different sensitivities. To test this hypothesis, we first replicated the collective failure reported by Bahrami *et al*. [9] (experiment 1: verbal condition). Then we devised a non-verbal confidence-rating/sharing schema to replace face-to-face verbal communication of decisions while keeping all other aspects of the experiment constant (experiment 1: non-verbal condition). If egocentric biases in face-to-face verbal communication were at least partially responsible for the collective failures in the verbal condition, then the non-verbal confidence rating/sharing schema employed in the non-verbal condition should improve collective performance under conditions of asymmetric sensitivity.

## 3. Experiment 1

### (a) Methods

#### (i) Participants

Participants were recruited from undergraduate, graduate and faculty members of Aarhus University, Denmark. Verbal (V) condition: *n* = 30, mean age ± s.d. 23 ± 2.5; non-verbal (NV) condition: *n* = 30, mean age ± s.d. 23.9 ± 2.5. All participants were healthy male adults with normal or corrected-to-normal visual acuity. Members of each dyad knew each other. No participant was recruited for more than one experiment. The local ethics committee approved all experiments, and written informed consent was obtained from all participants.

#### (ii) Display parameters and response mode

In all experiments, both dyad members sat in the same testing room. Each viewed his own display. Display screens were placed on separate tables at a right angle to each other. The two displays were connected to the same graphic card via a video amplifier splitter and controlled by the Cogent toolbox (http://www.vislab.ucl.ac.uk/cogent.php/) for Matlab (Mathworks Inc).

Each participant viewed an LCD display at a distance of approximately 57 cm (resolution = 800 × 600—Fujitsu Siemens AMILO SL 3220W, 22″) for which a look-up table linearized the output luminance. Background luminance was 62.5 Cd m^−2^ in both displays. The displays were connected to a personal computer through an output splitter that sent identical outputs to both of them. Within each session of the experiment, one participant responded with the keyboard and the other with the mouse. Both participants used their right hand to respond.

Each participant viewed one half of their screen: the left half of one display for the participant responding with the keyboard, and the right half of the other display for the participant responding with the mouse. A piece of thick black cardboard placed on each display was used to occlude the half not viewed by each participant. Two stimulus arrays were presented on both displays simultaneously, each on one-half of the display. Control over which one the participants saw was achieved by using the occluding cardboard. Stimulus eccentricity and retinal size were identical for both experiments. This configuration permitted us to display stimuli with different levels of noise to participants in the same dyad. Other stimulus characteristics (retinal size, luminance, contrast, duration) were identical for both participants. Moreover, in the NV condition, the use of a bipartite display allowed us to assess the participants’ confidence privately (i.e. each participants only saw his own confidence bar) at the individual decision stage (see §3*a*(v)).

#### (iii) Design and task

In all experiments, a two-Alternative Forced Choice (2AFC) design was employed (figure 2). Two observation intervals were provided. A target stimulus always occurred either in the first or in the second interval. Participants were instructed to choose the interval most likely to have contained the target. In the NV condition, participants rated their confidence in their decision on a scale from 1 (indicating ‘very doubtful’) to 5 (indicating ‘absolutely sure’; see below for a description of the confidence rating procedure and display).

#### (iv) Stimuli

The stimulus set displayed in each interval consisted of six vertically oriented Gabor patches (standard deviation of the Gaussian envelope: 0.45°; spatial frequency: 1.5 cycles deg^−1^; contrast: 10%) organized around an imaginary circle (radius: 8°) at equal distances from each other. The target stimulus was generated by elevating the contrast of one of the six patches, which produced a contrast oddball. The target location and interval were randomized across the experimental session. The stimulus duration in each interval was 85 ms. Target contrast was obtained by adding one of the four possible values (1.5%, 3.5%, 7.0% or 15%) to the 10% contrast of the non-target items.

For one participant, in each trial and for each item in the stimulus array, freshly generated white noise was added to the grey value of each pixel in each Gabor patch. The additional white noise was drawn, on each update, from a random uniform distribution ranging from 0 to 30% of the monitor's maximum luminance. The participants did not know about the addition of noise. The choice of which participant would receive the noise was determined by a preliminary test before the experiment (see below).

#### (v) Procedure

Each trial was initiated by the participant responding with the keyboard (figure 2). A black central fixation cross (width: 0.75° visual angle) appeared on the screen for a variable period, drawn uniformly from the range 500–1000 ms. The two observation intervals were separated by a blank display lasting 1000 ms. The fixation cross turned into a question mark after the second interval to prompt the participants to respond. The question mark stayed on the screen until both participants had responded. Each participant initially responded without consulting the other.

In the V condition, participants communicated by talking to each other. The participant who used the keyboard responded by pressing ‘N’ and ‘M’ for the first and second interval, respectively; the participant who used the mouse responded with a left and right click for the first and second interval, respectively. Individual decisions were then displayed on the monitor (figure 2), so both participants were informed about their own and their partner's choice of the target interval. Colour codes were used to denote keyboard (blue—illustrated in figure 2 by black) and mouse (yellow—illustrated in figure 2 by dark grey) responses. Vertical locations of the blue and yellow text were randomized to avoid spatial biasing. If the private decisions disagreed, a joint decision was requested. The request was made in blue if the keyboard participant was to announce the decision and in yellow if the mouse participant was to announce the decision. The keyboard participant announced the joint decision in odd trials; the mouse participant on even trials. Participants were free to verbally discuss their choice with each other as long as they wanted. They were also free to choose any strategy that they wished. The experimenter was present in the testing room throughout all experiments to make sure that the instructions were observed.

In the NV condition, participants did not talk to each other but instead used a visual schema (figure 2, lower panel) to communicate their confidence in their private decisions. After the two observation intervals, a horizontal line appeared on the screen with a fixed midpoint. The left side of the line represented the first interval, the right side of the line represented the second interval. An additional vertical ‘confidence marker’ (colour-coded for keyboard and mouse responses—see above) was displayed in each observer's panel. By dragging the confidence marker to the left or right from centre, the participant reported his choice about whether the target was in the first or second interval, respectively. The confidence marker could be moved along the line by up to five steps on either side. Each step farther from the centre indicated higher confidence. We chose this method for obtaining the decision (left or right side of centre) and the confidence in the decision (distance from the centre) all in one step rather than having the participants report them serially. This ensured that the participants’ private task involved only one step in all conditions here and in experiment 2. The participant who used the keyboard navigated the marker on the confidence rating scale by pressing ‘N’ or ‘M’ to move the marker left or right, respectively. He would then confirm his decision by pressing ‘B’ when he thought that the marker correctly indicated his confidence. The participant who used the mouse moved the confidence marker by pressing left or right button to move the marker left or right, respectively. He would then press the middle button when he thought the marker correctly indicated his confidence. Participants did not see each other's confidence rating at this stage. After the private confidence ratings were made, confidence values were announced by displaying both participants’ confidence markers along the horizontal line. In the case of disagreement, a joint decision was requested. Here, the keyboard participant announced the joint decision in odd trials, and the mouse participant on even trials. For the joint decision, a white confidence marker was used with the same five levels as private decisions; the marker was not visible to the other participant until a joint decision had been made. Participants did not talk to each other. They were given earphones to eliminate any meaningful auditory communication. In addition, a screen was placed between them to prevent them from seeing each other if they turned around. The experimenter was present in the testing room throughout all experiments to make sure that the instructions were observed.

In all conditions, participants received feedback either immediately after they made their private decision, in cases where their private decisions agreed, or after the joint decision had been made in cases where their private decisions disagreed. The feedback either said ‘CORRECT’ or ‘WRONG’. Feedback was given for each participant (keyboard: blue—illustrated in figure 2 by black; mouse: yellow—illustrated in figure 2 by grey) and for the dyad (white). Feedback remained on the screen until the participant using the keyboard initiated the next trial (figure 2). Vertical order of the blue and yellow was randomized and the dyad feedback always appeared at the centre.

In both conditions, participants started the experiment with a preliminary, non-interactive session (eight blocks of 16 trials) that was conducted in order to identify the participant who would receive noise in the subsequent main session (see §3*a*(vi)). Then, the main experimental session (eight blocks of 16 trials) was conducted.

#### (vi) Assignment of noise

We determined which participant would receive the noisy stimuli by first testing the participants in an isolated version of the task. In each trial, participants made a private decision about the target interval and then received private feedback (i.e. there was no sharing of private decisions and feedback). At the end of this session, the two participants’ sensitivity (i.e. the slope of the psychometric function; see §3*a*(vii)) was assessed, and the less-sensitive participant was chosen to receive the noisy stimuli in the experiment proper. The participants were not informed about this procedure and were told that the preliminary test served as practice.

#### (vii) Data analysis

**Figure 3.**
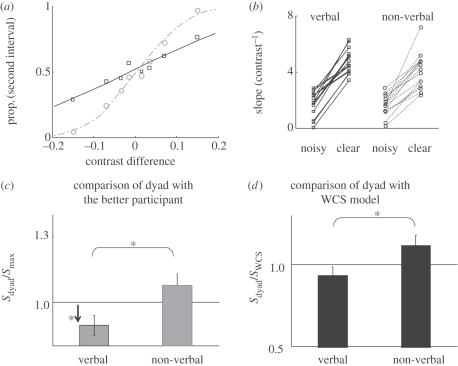
Results of experiment 1. (*a*) Psychometric function relating performance to contrast. Data are from the verbal condition of experiment 1 averaged across *n* = 15 participants for each curve. The proportion of trials in which the target was reported in the second interval is plotted against the contrast difference at the target location (i.e. contrast in the second interval minus contrast in the first). Participants who received clear stimuli (grey) produced steeply rising psychometric functions with large slopes. Participants who received noise (black) had a much shallower slope. (*b*) The slope of psychometric functions of the dyad members in the verbal and the non-verbal conditions of experiment 1. Each line corresponds to a dyad. Addition of noise was clearly effective at reducing the slope in both experiments. (*c*) Collective benefit (*s*_dyad_/*s*_max_; see §3*a*) accrued in the verbal and the non-verbal conditions of experiment 1. Horizontal line indicates that dyad slope was equal to the more sensitive participants. (*d*) Optimality of group performance in the verbal and the non-verbal conditions of experiment 1. Horizontal line indicates that group performance was as good as predicted by the WCS model (cf. Bahrami *et al*. [9]). *p < 0.05.

Psychometric functions were constructed for each observer and for the dyad by plotting the proportion of trials in which the oddball was seen in the second interval against the contrast difference at the oddball location (the contrast in the second interval minus the contrast in the first; figure 3*a*).

The psychometric curves were fit to a cumulative Gaussian function whose parameters were bias, *b*, and variance, *σ*^2^. To estimate these parameters, a probit regression model was employed using the ‘glmfit’ function in Matlab (Mathworks Inc). A participant with bias *b* and variance *σ*^2^ would have a psychometric curve, denoted *P*(*Δc*), where *Δc* is the contrast difference between the second and first presentations, given by3.1
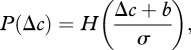


where *H*(*z*) is the cumulative normal function,3.2
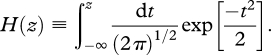


As usual, the psychometric curve, *P*(*Δc*), corresponds to the probability of saying that the second interval had the higher contrast. Thus, a positive bias indicates an increased probability of saying that the second interval had higher contrast (and thus corresponds to a negative mean for the underlying Gaussian distribution).

Given the above definitions for *P*(*Δc*), we see that the variance is related to the maximum slope of the psychometric curve, denote *s*, via3.3
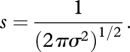


A large slope indicates small variance and thus highly sensitive performance. Using this measure, we quantified individual participants’ as well as the dyad's sensitivity. We defined ‘collective benefit’ as the ratio of the dyad's slope (*s*_dyad_) to that of the more sensitive observer (i.e. the one with higher slope, *s*_max_). A collective benefit value above 1 would indicate that the dyad managed to gain an advantage over its better observer. Values below 1 would indicate that collaboration was counterproductive and that the dyad did worse than its more sensitive member.

The WCS model expressed in equation (2.2) [9] identifies the dyad's *potential* for collective achievement under the assumption that the members can communicate their confidence to each other accurately. We compared the empirically obtained data with this potential upper bound to see whether and how different modes of communication helped or hindered collective decision-making. We defined an ‘optimality index’ as the ratio of the dyad's slope to that predicted by the WCS model (equation (2.2)).

### (b) Results

As demonstrated in figure 3*b*, all observers who received noise showed lower sensitivity (as measured by the slope of their psychometric function) compared with their partner who received noise-free stimuli. This result showed that our noise manipulation effectively rendered one participant's perceptual decisions much less reliable than those of the other. Under such conditions, the WCS model predicts that the dyad will do worse than the better observer.

#### (i) Comparison of dyad with the better participant

In the V condition, dyad sensitivity was significantly worse than that of the better observer (one sample *t*-test comparing collective benefit with baseline: *t*_14_ = −2.34, *p* = 0.03; figure 3*c*). This result is consistent with the predictions of the WCS model, which predicted that collective decision-making will be counterproductive when dyad members have very different sensitivities [9]. In the NV condition, on the other hand, dyad sensitivity was no worse than the more sensitive observer (one sample *t*-test comparing collective benefit with baseline: *t*_14_ = 1.42, *p* = 0.17; figure 3*c*) showing that groups had been at least as good as the better observer. Importantly, direct comparison of the two conditions showed that collective benefit was significantly greater in the NV condition (independent samples’ *t*-test: *t*_28_ = 2.61, *p* = 0.014).

#### (ii) Comparison of dyad with the Weighted Confidence Sharing model

The WCS model (equation (2.2)) slightly (but not significantly) overestimated dyad performance in the V condition (one sample *t*-test: *t*_14_ = 1.25, *p* = 0.23; figure 3*d*). In the NV condition, the WCS model showed a trend to underestimate the dyad performance (one sample *t*-test: *t*_14_ = 1.87, *p* = 0.08; figure 3*d*). Direct comparison of the two conditions showed that the optimality index was significantly higher in the NV condition (independent sample *t*-test: *t*_28_ = 2.24, *p* = 0.03).

### (c) Data summary

The impact of V and NV communication on collective decision-making was compared in an experimental situation, where a previous work had shown that dyads would perform no better than their constituting individuals [9]. The results replicate the previous findings but go beyond them in several respects: Bahrami *et al.* [9] had assigned noise to either one of the dyad members *at random.* This trial-by-trial random noise assignment made it impossible for the observers to form any stable idea of which dyad member was the less reliable one in any trial. Here we used a block design and assigned noise consistently to one member of the dyad. Thus, the results of the V condition (figure 3*c,d*) show an even more impressive collective failure: dyad sensitivity was significantly *worse* than the more sensitive dyad member. A conspicuous difference in performance did not protect the groups from suffering counterproductive collaboration.

The results of the NV condition showed that a non-verbal schema for reporting and sharing decision confidence (figure 1, lower panel), to some extent, could remedy the defective collective decision-making process and make it more productive; even though all the low-level conditions, especially the asymmetric administration of noise, were retained. This result is consistent with the suggestion that the collective failure observed in the V condition is not due to random errors caused by asymmetric noise but, rather, that direct, verbal communication and its associated underlying cognitive biases cause the collective failure.

**Figure 4.**
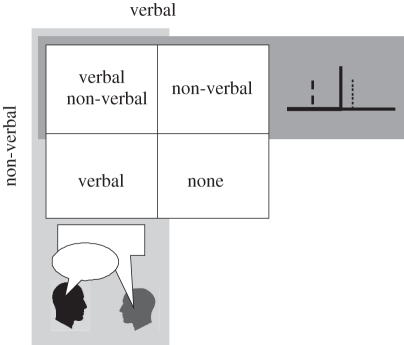
Two-by-two design employed in experiment 2.

Having demonstrated the beneficial impact of non-verbal communication on defective collective decisions, we asked whether this benefit is general. Experiment 1 tested collective decisions under asymmetric administration of noise where we expected no collective benefit to start with. When dyad members have access to similarly reliable perceptual information, however, direct verbal communication can indeed confer a robust group benefit that is no less than expected from the optimal combination of individuals’ decisions [9,10]. We asked whether the non-verbal communication of confidence could provide any additional benefit over and above direct verbal communication. Indeed, we do not know whether and how different modes of communication interact with one another towards collective decisions. In order to address this question, we used a 2 × 2 design where collective decision-making was tested under all four possible combinations of the two modes of communication (figure 4).

## 4. Experiment 2

### (a) Methods

#### (i) Participants

Participants were recruited from undergraduate, graduate and faculty members of Aarhus University, Denmark. Verbal&non-verbal (V&NV) conditions: *n* = 30, mean age ± s.d. 24.8 ± 3.5; verbal (V) condition: *n* = 30, mean age ± s.d. 28.30 ± 6.27; non-verbal (NV) condition: *n* = 30, mean age ± s.d. 22.2 ± 2; none (N) condition: *n* = 28, mean age ± s.d. 23.2 ± 2. All participants were healthy male adults with normal or corrected-to-normal visual acuity. Members of each dyad knew each other. No participant was recruited for more than one experiment. The local ethics committee approved all experiments and written informed consent was obtained from all participants. Data from the V and N conditions have been reported elsewhere [9].

#### (ii) Task and design

We employed a 2 × 2 design to investigate the impact of verbal communication (two levels: with and without) and non-verbal confidence sharing (two levels: with and without) on collective decision-making (figure 4). Participants communicated verbally in the V and V&NV conditions. In the NV and V&NV conditions, participants communicated using the confidence marker (as in the NV condition of experiment 1). In the none (N) condition, participants were not allowed to communicate anything but their decision (first or second interval). The task was identical to experiment 1 in all other aspects.

#### (iii) Display and stimuli

Participants received identical visual stimuli and no observer was given any additional noise. All stimulus characteristics were identical to the noise-free stimuli in experiment 1. In conditions that did not involve non-verbal communication (i.e. V and N conditions), the bipartite display was not used and both observers viewed a single stimulus set displayed at the centre of the entire screen. All other display and stimulus characteristics were identical to experiment 1.

#### (iv) Procedure

In all conditions, after one practice block of 16 trials, two main experimental sessions were conducted. Each main session consisted of eight blocks of 16 trials. Participants switched places (and thereby response device) at the end of session one. The participants set the pace of the experiment's progress. All other aspects of the procedure were identical to experiment 1.

### (b) Results

#### (i) Comparison of dyad with the better participant

**Figure 5.**
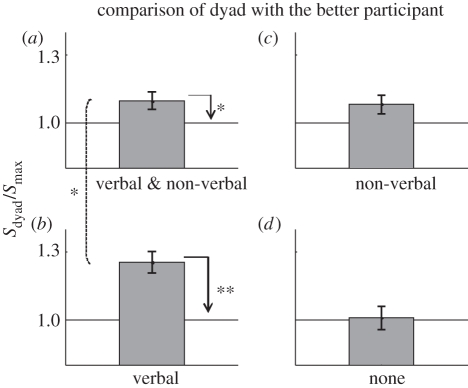
Collective benefit accrued in each condition of experiment 2. Panels correspond to the conditions illustrated in figure 3. Horizontal line indicates that dyad slope was equal to the more sensitive participants. *p < 0.05; **p < 0.01.

We first looked at the impact of the mode of communication on the collective benefit. Following our design (figure 4), we employed a 2 (with and without verbal communication) × 2 (with and without non-verbal confidence sharing) between-subject ANOVA with collective benefit (*s*_dyad_/*s*_max_; see experiment 1) as the dependent variable (figure 5). The main effect of verbal communication was highly significant (*F*_1,59_ = 8.4; *p* = 0.005). *Post hoc* comparison showed that collective benefit was significantly higher when verbal communication was allowed (i.e. conditions V and V&NV versus NV and N; independent sample *t*-test: *t*_57_ = 2.7, *p* = 0.008). Comparison with baseline (see horizontal lines in figure 5) showed that a robust collective benefit (i.e. group performance advantage over and above the better observer) was observed only where verbal communication is allowed, i.e. V&NV (figure 5*a*; one sample *t*-test: *t*_14_ = 2.47, *p* = 0.026) and V conditions (figure 5*b*; one sample *t*-test: *t*_14_ = 5.38, *p* < 0.0001). When communication was strictly non-verbal (NV condition), collective benefit marginally approached significance (figure 5*c*; one sample *t*-test: *t*_14_ = 2, *p* = 0.064). The main effect of non-verbal communication was not significant (*F* = 0.8). Finally, a significant interaction was found between verbal and non-verbal communication (*F*_1,59_ = 6.56; *p* = 0.013). *Post hoc* comparison showed that the interaction was driven by a significantly higher collective benefit in the V condition, where participants communicated *only* verbally; collective decision-making was significantly *less* successful when participants were required to use *both* verbal and non-verbal communication (i.e. V&NV versus V condition; independent sample *t*-test: *t*_28_ = 2.54, *p* = 0.016). Compared with no communication (N condition), the collective benefit accrued from non-verbal communication (NV) was not significant.

#### (ii) Comparison of dyad with the Weighted Confidence Sharing model

**Figure 6.**
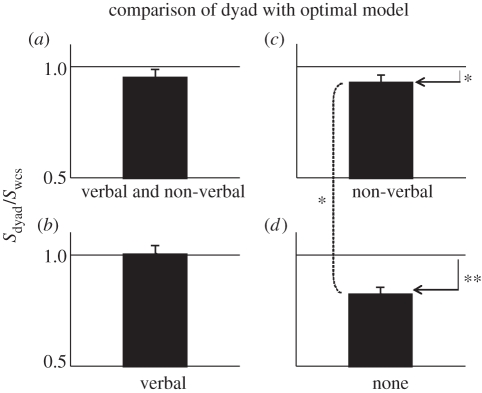
Optimality of dyad performance in each condition of experiment 2. Panels correspond to the conditions illustrated in figure 3. Horizontal line indicates that group performance was at the level predicted by the WCS model. *p < 0.05; **p < 0.01.

To compare the dyads’ collective performance with the upper bound set by the WCS model under different modes of communication, we applied a similar 2 × 2 repeated-measure ANOVA to the optimality index (figure 6). Similar to the collective-benefit analysis, the main effect of verbal communication (*F*_1,59_ = 8.745; *p* = 0.004) and the interaction between verbal communication and non-verbal communication *F*_1,59_ = 5.4; *p* = 0.024) was significant.

*Post hoc* comparison showed that the interaction was driven by the fact that sharing confidence non-verbally (NV condition; figure 6*c*) allowed the dyads to approach the WCS model significantly better than without any communication (N condition; figure 6*d*; independent sample *t*-test: *t*_27_ = 2.41, *p* = 0.02). This result demonstrated that although non-verbal confidence sharing is not an ideal mode of communication for collective decision-making (recall that the difference in collective benefit between the N and NV conditions was not significant), communicating ‘something’ is still better than ‘nothing’ for collective decision-making.

Comparison with baseline showed that when verbal communication was possible (V and V&NV conditions), dyads fulfilled the WCS model's expectations. With non-verbal communication only (figure 6*c*; *t*_14_ = 2.19, *p* = 0.04) and without any communication whatsoever, the model prediction exceeded the empirical dyad performance significantly (figure 6*d*; *t*_14_ = 5.91; *p* < 10^–4^, paired *t*-test).

### (c) Data summary

Because dyad members received identical visual stimuli without any asymmetric noise, collective benefit was expected in all communicative conditions (except the N condition). Collective benefit was robustly obtained when participants communicated only verbally. Non-verbal communication alone (NV condition) also showed some benefit: dyad performance was closer to the optimal upper bound (predicted by the model) than no communication (N condition; figure 6) and a trend was observed for collective benefit (figure 5). Surprisingly, when dyad members communicated by both means, they obtained less benefit than when they communicated only verbally (figure 5*a* versus *b*). The benefits of verbal and non-verbal communication were, so to speak, sub-additive.

## 5. Discussion

‘*How can we aggregate information possessed by individuals to make the best decisions?*’ Condorcet, Galton and Mackay would have been pleased (or disappointed?) to know that a recent survey (http://bit.ly/hR3hcS) of current academic opinions has listed this question as one of the 10 most important issues facing social sciences in the twenty-first century. The data presented here directly address this question and the results provide recommendations for enhancing the accuracy of collective decisions under different circumstances.

Experiment 1 showed that the success of collective decision-making is severely compromised if the quality of evidence available to verbally communicating collaborators is very different. When participants could communicate verbally, asymmetric sensitivity of the team members led to counterproductive collaboration, even though block-design administration of noise to one member, but not the other, caused a striking and persistent difference in the outcome accuracy between the two collaborators. These results delineate a critical danger facing collective decisions: too wide a competence (i.e. in our case, perceptual sensitivity) gap among interacting agents leads to collaborative failure even if the gap is conspicuously obvious. If we needed any quantitative evidence for the ‘madness of the crowds’, this could be it. However, when participants could only share their confidence non-verbally, dyads did significantly better than those who had talked to each other directly, even though the competence gap was still firmly in place. The latter findings suggest that groups composed of members with very different competences could avoid major losses (and perhaps even accrue some collaborative benefit) if a suitable mode of communication was adopted.

This result is consistent with the ‘egocentric bias’ hypothesis from earlier work [14] suggesting that verbally interacting human agents operate under the assumption that their collaborators’ decisions and opinions share the same level of reliability. As long as this assumption holds (i.e. *s*_Q_ ≈ *s*_J_), verbal communication provides an efficient strategy for aggregating information across individuals and making decisions that are as good as if the individuals had direct access to each other's mental representations (cf. comparison of equations (2.1) and (2.2)). However, verbal communication backfires when the egocentric assumption does not hold (e.g. *s*_Q_ ≫ *s*_J_). An important question for future research is to see what aspect of verbal communication is responsible for upholding the egocentric bias despite recurring collective failure. Social obligation to treat others as equal to oneself (despite their conspicuous inadequacy) or the urge to contribute (or *make a difference*) to the group—despite objectively proving to be less competent—are two candidate mechanisms.

In experiment 2, in a 2 × 2 design (figure 3), we systematically investigated the impact of verbal communication and non-verbal confidence sharing on collective decision-making. The results showed that combining the two modes of communication was counterproductive. When group members had similar sensitivity and made decisions based on similarly reliable information, best (near optimal) performance was achieved with direct verbal communication. Imposing an additional non-verbal communication tool significantly reduced the group performance. Collective benefit was, so to speak, *crowded out*.

A plausible explanation for this observation may be found in the literature on introspection and metacognition. The confidence-rating schema required participants to actively introspect about their perceptual experience and then graphically indicate their internal, metacognitive estimate of the reliability of their decision (i.e. their confidence). This is no mean feat and indeed a costly cognitive task that requires allocation of top-down attention [17,18]. In the condition where participants both communicated verbally and used the confidence-rating schema, it is conceivable that the cognitive load introduced by active introspection may have interfered with the verbal communication and the collective decision-making process. Unconstrained verbal communication is perhaps more automatic and humans may be thought of as ‘natural experts’ in it. Indeed, a recent study [19] (see also Overgaard and Sandberg, this issue; [20]) has suggested that asking people directly about their perceptual experience, rather than having them rate their confidence, may give a more accurate measure of their metacognitive awareness. Future research could show if more practice with active confidence rating could lead to more automatic, effortless introspection, which in turn might contribute to enhanced collective decision-making beyond what is achievable by direct verbal communication.

**Figure 7.**
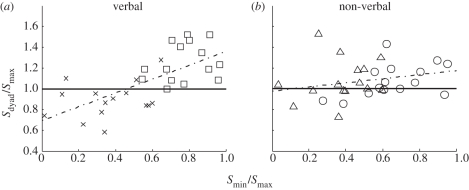
The relationship between collective decisions and similarity of dyad members’ sensitivity. Each panel shows the relationship between collective benefit (*s*_dyad_/*s*_max_) and similarity (*s*_min_/*s*_max_; see §4*a*). (*a*) Data from experiments with exclusive verbal communication mode (cross symbols: verbal condition in experiment 1; squares: verbal condition in experiment 2). (*b*) Data from experiments with exclusive non-verbal communication mode (triangles: non-verbal condition in experiment 1; circles: non-verbal condition in experiment 2).

When we consider experiments 1 and 2 together, an intriguing crossover effect is observed: in experiment 1 (with unequal external noise) collective benefit was higher when participants only share confidence non-verbally, whereas in experiment 2 (with equal external noise), verbal communication was clearly superior. The crossover is not consistent with any explanation relying on a single mechanism to determine the success of collective benefit. Egocentric bias inherent in verbal communication (experiment 1) and cognitive load of introspection (experiment 2) may be interacting with one another to give rise to this crossover. This suggests that the preferred mode of communication of confidence for collective decisions depends on the *similarity* of dyad members’ sensitivity. This idea is illustrated in figure 7. Each panel shows the relationship between similarity (*s*_min_/*s*_max_) and collective benefit (*s*_dyad_/*s*_max_). Data are from parts of experiments 1 and 2 in which communication was exclusively verbal or exclusively non-verbal. The instructive conclusions for how to maximize collective benefit are clear: when dyad members are highly similar (*s*_min_/*s*_max_ > 0.6), direct verbal communication should be used (squares in figure 7*a*). But the substantial benefit from verbal, direct engagement strongly depends on the similarity of dyad members’ competence. When observers have very dissimilar sensitivities—*s*_min_/*s*_max_ < 0.6—direct communication is disastrous (cross symbols in figure 7*a*). In such situations, non-verbal confidence sharing communication (triangles in figure 7*b*) is recommended: it could save the dyad by avoiding the counterproductive collaboration that is observed with direct verbal communication.

These results provide algorithmic guidelines for Qodrat and Jalal (figure 1) in their effort to maximize the accuracy of their perceptual judgement as a group of umpires. However, perhaps with the exception of refereeing in sports games, collective decisions are rarely about purely perceptual events amidst uncertainty and noise. In the next section, we will discuss other domains of social interaction where collective failures have been reported and compare them with these findings.

## 6. Collective failures in non-perceptual domains

Numerous studies in social psychology have documented instances where group performance is worse than the performance of the best member. In social loafing [21], individuals exert less effort in the presence of others leading to reduced overall group performance. Thus, social loafing refers to the difference in individual performance when individuals act in isolation versus when they act together as a group. An important feature of collective situations in which social loafing has been observed (e.g. the ‘tug of war’ game) is that group members share the responsibility for possible failures such that no specific member could be singled out and held directly responsible for the group's misfortune [22].

The collective failures described here and by Bahrami *et al*. [9] are different from social loafing for two reasons. First, here dyad members were always tested in the presence of their partner. The social setup of the task was identical for dyads that consisted of similarly sensitive members (who achieved a collective benefit) and those with dissimilarly sensitive members (who incurred a collective loss). In none of the conditions discussed above did the participants perform the task ‘in isolation’. Even when participants did not communicate either verbally or non-verbally (figures 3, 5*d* and 6*d*), they were still sitting in the same room and shared decisions and made joint decisions when in disagreement. Interestingly, a recent finding has suggested that individual sensitivity assessed in collaborative settings (i.e. private decision stage; figure 2) was superior to individual sensitivity assessed in non-collaborating setting where two observers were independently tested simultaneously in the same room [23]. This individual sensitivity advantage required the dyad to actively engage in the joint decision-making and was therefore different from social facilitation induced by the mere inactive presence of another person [24]. Second, in all experiments described here, decision outcomes were clearly stated for the group as well as both participants leaving little room for sharing the responsibility for group failures. The participant who led the group to the wrong decision had, so to speak, nowhere to hide. This is an important feature of these experiments which shields the group performance against motivation loss [25].

Groupthink [26] is another case of collective failure. When individuals are not given the opportunity to make their own decisions privately, they subsequently fail to develop and voice their disagreeing opinions. Interdependence of individual decisions leads to groupthink [27]. This phenomenon cannot account for the results reported by Bahrami *et al*. [9] because individual decisions were always first made privately and independently.

Interpersonal competition [28] is also ruled out as the observers were not differentially rewarded for their decisions and there was no incentive for competition.

Finally, the hidden profile paradigm [29–31] is another extensively studied case of collective failure with interesting similarities with and differences from the cases discussed so far. In 1985, Stasser & Titus [29] discovered that group interactions tend to focus on information shared by everybody. This even happens when some of the interacting individuals have access to unshared information that is fundamentally relevant—and provides the best solution—to the joint decision problem and it is in the interest of all individuals to share that exclusive information. In other words, group interactions are biased away from hidden profiles. Groups composed of members with dissimilar knowledge profiles thus tend to under-exploit their unshared but available and relevant information.^3^

The pattern of collective behaviour in hidden profile paradigm is consistent with the illusion of transparency [15] and the egocentric biases [16] in interpersonal communication. Indeed, the verbal condition in experiment 1—where one person is much better than the other (*s*_max_ ≫ *s*_min_)—may involve a similar situation: the better person might be seen as having some implicit knowledge (e.g. less noisy stimulus) that the other does not have. However, it is difficult to explain the collective failures that were exposed here based on hidden profile paradigm *per se*. The marked difference in participants’ accuracy on a trial-by-trial basis was common knowledge because the feedback was given to both individuals at the same time. As such, after a few trials, the asymmetric reliability of the observers in experiment 1 was not exclusive knowledge at all. Moreover, the detrimental impact of asymmetric noise on collective performance was observed only when dyads communicated directly rather than when they shared confidence using the visual schema. If the hidden profile paradigm was responsible for the collective failures, one would expect not less but, rather, maybe even more collective failure when communication was minimized and only non-verbal confidence sharing was used. Nonetheless, it is possible that verbal communication masks the sensitivity gap, whereas non-verbal communication strips away the social interaction and magnifies the gap, making it easier to discard the less-sensitive participant's opinion. At present, the only firm conclusion on this issue would be that more research is needed to address these possibilities.

## 7. The impact of interaction on alignment of metacognition

What are the qualitative features of sharing and discussing metacognitive awareness when Qodrat and Jalal (figure 1) discuss their opinions? Recently, we have undertaken linguistic analysis of the conversations leading to the collective decisions in the verbal condition of experiment 2 [32]. The results (not reported here) showed that dyadic conversations often focus on participants’ confidence in their decisions. Most groups used more everyday expressions such as ‘*I was not so sure*’ and ‘*I saw it clearly*’. The conversations rarely (i.e. 1 out of 30 sessions studied) led to spontaneous use of explicit numerical scales to express and compare confidence. On a trial-by-trial basis, interacting observers tended to align with each other's confidence expressions. For example, if one started the conversation with ‘I did not see anything’, the other person would most likely respond with some expression using ‘see’. Over time, the content of conversations tended to diminish with practice such that by the end of the experiment, dyad members had converged to a small, repeatedly used set of expressions.

**Figure 8.**
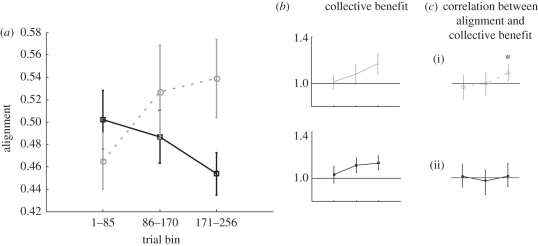
(*a*) Alignment of confidence plotted for each time bin consisting of one-third of the trials. Black symbols and curve correspond to the verbal and non-verbal condition in experiment 2 where both participants communicated verbally and used the confidence rating schema. Grey symbols and curve correspond to the non-verbal condition in experiment 2, where participants only communicated via the confidence rating schema. Error bars are 1 s.e.m. across dyads (*n* = 15). (*b*) Collective benefit (*s*_dyad_/*s*_max_) is plotted for each bin. Horizontal line indicates no benefit (i.e. *s*_dyad_ = *s*_max_). Error bars are 1 s.e.m. across dyads (*n* = 15). (*c*) Correlation coefficients between alignment and collective benefit across dyads for each time bin. For V&NV condition (ii), (Pearson *r* = [0.08, −0.15, 0.11] and all *p* > 0.55. For NV condition (i), Pearson *r* = [0.1, −0.01, 0.6] and *p* = [0.7, 0.9, 0.01]. Horizontal line indicated zero. Departure from null hypothesis (*p* = 0.01) is marked by asterisk. Error bars are 95% CIs for Pearson correlation using Fisher transformation [33] (http://bit.ly/pvDx9).

These qualitative and quantitative observations [32] prompted us to wonder if a similar practice-dependent alignment of confidence could be observed in the conditions of experiment 2, where the confidence rating schema was employed. To test this hypothesis, we revisited the data from the NV and V&NV conditions of experiment 2. For each trial, we calculated the absolute difference in signed confidence rating (see §4*a*) between the two observers and defined alignment as the inverse of this difference. The results (figure 8) showed robust evidence for increased alignment in the NV condition where participants used only non-verbal confidence sharing (figure 8*a*, grey: one-way ANOVA with three levels for first, middle and last one-third of trials, *F*_2,28_ = 5.38, *p* < 0.012). These results are in line with the qualitative findings about linguistic alignment of confidence [32] in the verbal condition of experiment 2 and show that as dyad members gain experience from their interactions, they tend to ‘describe’ their confidence more similarly using the confidence rating bar.

However, in the V&NV condition of experiment 2 where participants used both verbal and non-verbal communication, confidence alignment decreased (figure 8*a*, black: one-way ANOVA, *F*_2,28_ = 5.16, *p* < 0.013). In other words, confidence judgements diverged from one another over time. Although use of the confidence rating schema alone led to alignment of observers' metacognitive reports, combining verbal communication and non-verbal confidence ratings led to a divergence of confidence ratings.

Direct comparison of the NV and V&NV conditions using a mixed ANOVA (with two levels for conditions and three levels for trial bins) supported this conclusion with a significant interaction (*F*_2,56_ = 9.34, *p* < 0.0001). These results corroborate the idea suggested earlier that combining both modes of communication—as in the V&NV condition—leads to an interference in task performance both at the individual (as we see here) and at the collective level (as we saw earlier in the results of experiment 2).

Does alignment of metacognition have any relevance for collective decision-making? In the conditions of experiment 2 where participants rated their confidence, for each dyad, we calculated the collective benefit accrued within each one-third of the experiment (figure 8*b*). We then tested whether there was any correlation between alignment (figure 8*a*) and collective benefit (figure 8*b*) across the dyads. The results showed that a significant correlation (Pearson *r* = 0.6, *p* = 0.01, *n* = 15) emerged in the last third of the NV condition (figure 8*c*(i)). When participants shared only confidence rating but did not talk, metacognitive alignment was associated with collective benefit suggesting that with enough practice, dyad members may arrive at and use the alignment to make better group decisions. In line with our previous observations, in the V&NV condition (figure 8*c*(ii)) where participants both talked and used the confidence rating schema, no such relationship ever emerged. This finding once again underscores our conclusion that the combination of both modes of communication was not productive. Moreover, the fact that the overall collective benefit was not statistically different between the V&NV and the NV conditions (figure 5*a*,*c*) suggests that dyads in these two conditions achieved the same level of performance employing different strategies for communication and decision rules. More research is needed to understand the nature of these different strategies and decision rules.

We also conducted a similar alignment analysis on the data from the NV condition of experiment 1. Our results did not show any significant findings either for the alignment of confidence ratings or for a correlation between alignment and collective benefit. We believe that this is most likely owing to the fact that the number of trials in the interactive phase of experiment 1 (i.e. 128) was half that of experiment 2; remember that the effects that we see in figure 8 did not emerge before the final third of the trials (171–256). Aside from statistical power issues, however, we are also reluctant to make a strong prediction about alignment of confidences in experiment 1 as the main manipulation in that experiment was to deliver different and uncorrelated levels of independently generated visual input noise to the two participants in each trial, which is expected to weaken any developing/existing correlation among any outputs from the two observers including confidence ratings.

## 8. The role of feedback and the contribution of shared metacognition to social learning

Could Qodrat and Jalal (figure 1) achieve any collective benefit from sharing their opinions and discussing their disagreements if they never found out who was actually right and who was wrong? This is an important question because none of the information integration models that we have discussed here [9,10] assume any role for decision outcomes. Moreover, and perhaps more importantly, both models attempt to explain the dyad behaviour as a stable, stationary phenomenon with little variability over time. As useful as these assumptions may be for simplifying the problem, few would agree that human group behaviour is—in general—independent of outcomes and unaffected by social learning.

A recent study [34] examined the development of collective benefit when feedback was withdrawn from the dyads. Without feedback, dyads did not initially achieve any collective benefit. However, with practice dyads started to exceed their more sensitive member such that by the end of the experiment, the collective benefit of interacting dyads with and without feedback was statistically indistinguishable. Thus, knowledge about outcomes only seemed to accelerate the process of social learning required for efficient confidence sharing.

Interestingly, feedback is not necessary for optimal multisensory integration of visual and haptic information [35]. Following the standard practice in psychophysics, those results were obtained from several thousands of experimental trials for each observer to make sure that performance is measured long after any learning process is finished. It is, therefore, likely that feedback plays a similar accelerating role in achieving optimality in multisensory integration. To our knowledge, previous research in perceptual learning of multisensory integration has not addressed the role of outcome information on the speed of learning.

Once again, collective failure is instructive in helping us to phrase the right research question. The initial failure of the no-feedback groups to exceed their best member and their subsequent improvements to the same level as feedback groups pose serious problems for models of collective decision-making that assume no social learning [9,10]. An important question for future research is to explain the dynamics of social learning needed to achieve effective collective behaviour over the course of repeated interactions in the absence of feedback.

Currently, a number of computational models have been proposed for social learning based on principles of associative reinforcement learning [36–38]. The critical question here is: how could dyad members in the no-feedback experiment [34] have accomplished reinforcement learning without any reinforcement (i.e. without knowing the outcome of their decisions)? It has been suggested [34] that sharing metacognitive awareness may provide sufficient information to replace feedback and reinforce social learning. On this account, when participants are sincere in their opinions, the shared metacognitive awareness that informs the joint decision provides a noisy but still informative estimate of the true state of the world which can be used as a substitute for the missing feedback about decision outcomes [39]. With a noisy substitute, the reinforcement learning process could still happen but would take longer to develop. With enough practice, learning with and without feedback would eventually stabilize at similar performance levels. This account [34] has an interesting, if unexpected corollary: a functional role of shared metacognitive awareness may be to replace missing reinforcement signals when decision outcomes are not available (e.g. too complex to estimate or too far in the future to wait for). Given the abundance of situations in everyday life where immediate outcomes are difficult, sometimes even impossible, to establish, the hypothesis proposed by Bahrami *et al*. [34] offers an ecologically relevant role for metacognition.

## 9. Neuronal, behavioural and social metacognition

Historically, decision science has focused on three aspects of every decision: accuracy, reaction time and confidence [40,41] often assuming that all three originate from the same underlying process [42]. The *sequential sampling* family of models [43] was developed to account for speed-accuracy trade-offs observed in two-alternative choice tasks (for a review see Kepecs & Mainen [44]). The idea in sequential sampling is that when an observer is presented with some sensory signal and asked to categorize it as A or B, s/he keeps sampling the signal and accumulating the evidence for each alternative. The race between the two accumulators goes on until evidence collected for one category hits a predefined boundary determining the chosen category for the signal. These three components, a sensory receptor, an accumulator and a boundary are the backbone of perhaps the most widely popular decision-making models in today's system neuroscience [45,46].

Sequential sampling models have been extended to account for decision confidence as the difference in accumulated evidence supporting each category at the decision time [47]. Heath [48] showed that such a ‘balance of evidence’ concept can account for a number of qualitative features of decision confidence [48]. Recent works have found neuronal substrates in rodent [49] and non-human primate [50] brains for decision confidence that closely overlap with the known neural machinery involved in decision accuracy and speed. Moreover, the firing patterns of these confidence neurons closely match the predictions of the sequential sampling models. These latter findings thus provide evidence for the earlier intuition that decision accuracy, reaction time and confidence arise from the same latent neuronal process [40]. As such, neuronal encoding of confidence seems to be the cost-free, automatic by-product of the decision process.

But this view is hardly consistent with what is known about metacognition at the level of behaviour. Introspection is cognitively demanding [17] and therefore neither automatic nor cost-free. Moreover, if the confidence and choice processes were one and the same, then restriction of choice time should systematically reduce metacognitive accuracy in a manner parallel to standard speed-accuracy trade-offs. However, when speed is stressed in a choice reaction time task, choice accuracy decreases as expected but, paradoxically, metacognitive accuracy increases [51]. This suggests that rating confidence may involve some post-decisional processing distinct from the race-to-boundary stage (also see Yeung & Summerfield [52]). A tantalizing prediction arising from this notion is that, if one repeats our experiments (reported above) with an emphasis on speed (rather than accuracy) in the initial perceptual task, then sharing (supposedly) more accurate metacognitive awareness should enhance the collective benefit.

But the data we have reported here (experiments 1 and 2) caution against tightly connecting behavioural metacognition with shared, social metacognition. Our results showed that effective sharing of metacognitive awareness depends on some form of social heuristics (e.g. egocentric bias) and that the sharing process seems to be dissociable from and interact with the cognitive demands of introspection, i.e. behavioural metacognition. As such, our understanding of metacognition at the levels of neuronal representation, behaviour and social interaction seem to be disconnected at the moment calling for future research to (see if it is possible or meaningful to) bring them together.

Finally, the concepts of confidence in perceptual sciences and *uncertainty* in neuroeconomics both refer to the subjective probability of choice outcomes. Over the past decade, many studies in the field of neuroeconomics [53] have decomposed the notion of uncertainty into ‘risk’ and ‘ambiguity’ and demonstrated the behavioural and neurobiological correlates of each [54–56]. When the possible outcomes and their respective probabilities are known, the decision is said to be risky. Ambiguity, on the other hand, refers to situations where the outcomes alternatives and/or their respective probabilities are unknown. At first glance, confidence in a perceptual two-alternative forced-choice judgement in the lonely darkness of a psychophysics laboratory may seem to be closer to the concept of risk. However, a conspicuous distinction here seems to be that risk and ambiguity both refer to subjective probabilities (or their lack of) *prior to* choice, whereas confidence refers to subjective probabilities that arise *during* evidence accumulation and *after* the choice has been made. Moreover, whereas perceptual confidence depends critically on internal (neural) and external (environmental) sources of noise, previous works in neuroeconomics have not used noisy sensory information to manipulate risk or ambiguity and therefore the connection between these inter-related concepts is at present unclear but indeed a very interesting topic for future research that is just beginning to be investigated [57].

## 10. Closing remarks

Two heads are not always better than one. This paper focused on recent models and empirical findings that explored collective failures. These models are inspired by thinking of collective decision-making as an ‘information integration’ problem similar to that of multisensory perception. The intuition obtained from these theoretical and empirical findings is that shared metacognitive awareness (socially communicated confidence in one's own perceptual decisions that contributes to collective perceptual decisions) conveys the strength of the sensory experience and its reliability inseparably. An important functional role of such metacognitive awareness may be to substitute missing outcomes in situations where outcome is necessary for learning but unavailable or impossible to establish.

## Endnotes

1 Interestingly, his major case studies, financial bubbles and religious conflicts over Jerusalem, do not show any signs of running out of steam yet.

2 The names chosen for the cricket umpires are inspired by Graham Greene's [12] *The Power and the Glory* (Qodrat and Jalal).

3 Thomas Bayes (http://bit.ly/f0uTBk) would perhaps have found the bias for favouring redundant and frequent information only wise and sensible. In the words of Bellman in Lewis Carrol's brilliant *The Hunting of the Snark*, ‘JUST the place for a Snark!’ the Bellman cried, As he landed his crew with care; Supporting each man on the top of the tide By a finger entwined in his hair. ‘Just the place for a Snark! I have said it twice: That alone should encourage the crew. Just the place for a Snark! I have said it thrice: *What I tell you three times is true.*’
